# Differentiation of Developmental Pathways Results in Different Life-History Patterns between the High and Low Latitudinal Populations in the Asian Corn Borer

**DOI:** 10.3390/insects13111026

**Published:** 2022-11-06

**Authors:** Shu Fu, Lili Huang, Haimin He, Jianjun Tang, Shaohui Wu, Fangsen Xue

**Affiliations:** 1 College of Oceanology and Food Science, Quanzhou Normal University, Quanzhou 362000, China; 2 Institute of Entomology, Jiangxi Agricultural University, Nanchang 330045, China; 3 Department of Ecology and Environment, Yuzhang Normal University, Nanchang 330103, China; 4 College of Computer and Information Engineering, Jiangxi Agricultural University, Nanchang 330045, China; 5 Department of Entomology, University of Georgia, Tifton, GA 31793, USA

**Keywords:** *Ostrinia furnacalis*, body weight, developmental time, growth rate, non-diapause, diapause

## Abstract

**Simple Summary:**

The Asian corn borer, *Ostrinia furnacalis* (Guenée), is geographically widespread and enters a photoperiod-induced larval diapause with the gradually shortening daylengths of autumn. Here, life-history traits between non-diapausing and diapausing individuals were tested along a latitudinal gradient by rearing the larvae from six different geographical populations under their own critical daylengths to produce non-diapausing and diapausing individuals. In the non-diapausing pathway, the high-latitudinal populations had a significantly shorter larval developmental time and greater body weight than the low-latitudinal populations, whereas in the diapausing pathway, the high-latitudinal populations had longer larval developmental times and relatively smaller body weights than the low-latitudinal populations. This is the first report showing that life-history patterns of the two alternative developmental pathways were significantly different between the lower and higher latitudes.

**Abstract:**

Individual insects often exhibit two alternative pathways of non-diapausing and diapausing developments. Yet, most studies have focused on the latitudinal variation in life-history traits for non-diapausing individuals. No study has examined the differences in life history traits between non-diapausing and diapausing individuals along a latitudinal gradient. We used six different geographical populations of *Ostrinia furnacalis* to examine the latitudinal variation in life-history traits between non-diapausing and diapausing individuals in terms of their sex ratio, larval and pupal developmental times, pupal weight, growth rate, adult weight and weight loss, and sexual size dimorphism. The results showed that latitudinal variation in life-history traits for both non-diapausing and diapausing individuals exhibited a sawtooth pattern, but the life-history pattern of the two alternative developmental pathways was significantly different between the high and low latitudes. For the non-diapausing pathway, the high-latitudinal populations showed a significantly shorter larval developmental time, higher growth rate and greater body weight than the low-latitudinal populations, suggesting countergradient variation. Conversely, in the diapausing pathway, the high-latitudinal populations had longer larval developmental times, lower growth rates and relatively smaller body weights than the low-latitudinal populations, suggesting cogradient variation. We also found that in the high-latitudinal populations, larvae in the non-diapausing pathway had shorter developmental time and higher body weight, whereas larval developmental time of the low-latitudinal populations was longer and the body weight was smaller. The relationship between larval developmental time and pupal weight was also different between the two developmental pathways. These results provide new insights into the evolution of life-history traits in this moth.

## 1. Introduction

Organisms have evolved different strategies to meet the challenge of environmental variations, including genetic adaptation and phenotypic plasticity [1,2,3,4]. Developmental plasticity is one of the main forms of adaptation to environmental changes and plays a central role in biodiversity [5]. Insect diapause is a classic and famous example of developmental plasticity. Many insect species, such as the cabbage butterfly, *Pieris melete* [6], the zygaenid moth, *Pidorus euchromioides* [7], and the cabbage beetle, *Colaphellus bowringi* [8], are known to enter part of their population into diapause, while the rest continue to develop and reproduce when they are reared under constant laboratory conditions or experience the same field conditions. Especially in insects with photoperiodic induction of diapause, both non-diapausing and diapausing developments are often observed when the rearing daylength is close to critical daylength (the daylength that elicits 50% diapause or development response). This plasticity of diapause incidence was described as “bet-hedging” or “dispersive breeding” strategies against unpredictable risks due to fluctuating environmental conditions [9,10]. Individual insects that demonstrate non-diapausing and diapausing developments are usually described as presenting alternative developmental pathways [11,12,13]. However, most studies have concentrated on the latitudinal variation in life-history traits for non-diapausing individuals [14,15,16,17,18,19,20,21,22,23,24]. Several studies have examined the differences in life history traits between non-diapausing and diapausing individuals, such as the striped ground cricket, *Allonemobius fasciatus* [25], the striped ground cricket, *Allonemobius fasciatus* [26], the butterfly, *Pieris napi* [27], and the cabbage butterfly, *Pieris melete* [28], in which diapausing individuals were larger than non-diapausing ones. However, in the moth *Cabera exanthemata*, non-diapausing individuals had shorter developmental times, higher growth rates and higher pupal masses than individuals entering diapause [29].

Species with broad geographic ranges often exhibit latitudinal gradients in climate. It is probably to be expected that latitudinal gradients in climate may translate to biological gradients in some life-history traits. It has been reported that 123 studies for insects showed Bergmann clines (with larger individuals at higher latitudes), and 111 studies showed converse Bergmann clines (with smaller individuals at higher latitudes) [30]. Developmental time and growth rate may be cogradient variations, with low-latitude populations having a faster intrinsic growth and developmental rate than those from higher latitudes, or countergradient variations, with high-latitude populations having a faster intrinsic growth and developmental rate than those from lower latitudes [23,31]. Conover et al. [31] reported examples of gradient variations from the literature and discovered 13 insect species showing countergradient variation and only two insect species showing cogradient variation. Recently, six species have shown concrete evidence of countergradient variation [22,24,32], and two species displayed cogradient variation [23,33]. However, for some lepidopterous insects, latitudinal size variation has not been found [19,23,34]. Furthermore, size patterns wholly or partly following a saw-tooth pattern (with peaks and troughs but no clear trends between size and latitude) have been described in some species [19,25,35,36,37,38].

The Asian corn borer, *Ostrinia furnacalis* (Guenée) (Lepidoptera: Crambidae), is geographically widespread and enters a photoperiod-induced larval diapause with the gradual shortening daylengths in autumn [39,40]. This species displays considerable diversity in life history between different geographical populations. Populations differ significantly in generation number, critical daylengths [41,42], postdiapause emergence time [43], cold hardiness [44] and life-history traits [34]. Thus, the moth provides an ideal experimental model to record its geographical variation in life-history traits between non-diapausing and diapausing individuals.

In this study, we compared the life-history traits between non-diapausing and diapausing individuals along a latitudinal gradient (from 29°04′ N to 44°93′ N) by rearing the larvae from six different geographical populations under their own critical daylengths to produce both non-diapausing and diapausing individuals. Specifically, we examined the sex ratio, larval and pupal developmental times, pupal weight, growth rate, adult weight and weight loss, and sexual size dimorphism for both non-diapausing and diapausing individuals in each population. To date, no study has compared the life-history traits between non-diapausing and diapausing individuals along a latitudinal gradient. This study will broaden our understanding of changes in life-history traits associated with different developmental pathways across a geographical range.

## 2. Materials and Methods

### 2.1. Study Populations and Insect Culture

**Figure 1:**
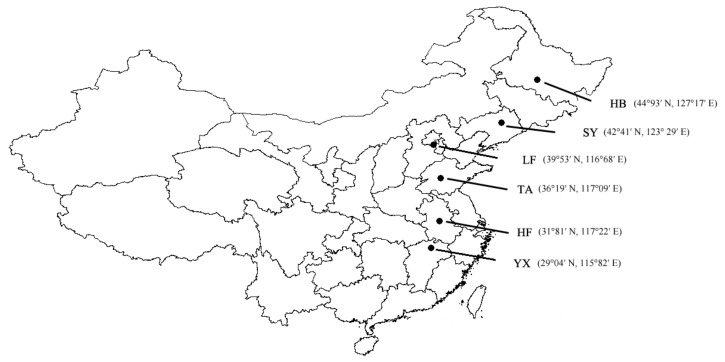
The collection sites of samples of *Ostrinia furnacalis*. HB: Harbin city, Heilongjiang Province; SY: Shenyang city, Liaoning Province; LF: Langfang city, Hebei Province; TA: Taian city, Shandong Province; HF: Hefei city, Anhui Province; YX: Yongxiu County, Jiangxi Province.

Adult females were netted from corn fields with a net from six regions, i.e., Yongxiu County (29°04′ N, 115°82′ E), Jiangxi Province (as the YX population); Hefei city (31°81′ N, 117°22′ E), Anhui Province (as the HF population); Taian city (36°19′ N, 117°09′ E), Shandong Province (as the TA population); Langfang city (39°53′ N, 116°68′ E), Hebei Province (as the LF population); Shenyang city (42°41′ N, 123°29′ E), Liaoning Province (as the SY population); and Harbin city (44°93′ N, 127°17′ E), Heilongjiang Province (as the HB population) (Figure 1). Wild-caught females (30–40 females per population) were put into plastic bags with 10% honey-water to lay eggs. Egg masses were gathered and put in Petri dishes. When larvae hatched, they were transferred to plastic boxes (diameter 12 cm, height 15 cm) and were fed artificial diets [45] under a long photoperiod of L:D 18:6 (18 h light: 6 h dark) at 25 °C until adult eclosion. Newly emerged moths were transferred into cages made from freshness-protection bags (20 × 30 cm) inflated with air to mate and produce egg masses. A cotton ball moistened with 10% honey water was placed in a plastic bag to nourish adults. Egg masses produced in the first three days were used for experiments.

### 2.2. Experimental Methods

The newly hatched larvae from the six populations were moved to plastic boxes (diameter: 12 cm; height: 15 cm) and were fed artificial diets. The plastic boxes were placed in illumination incubators (LRH-250-GS, Guangdong Medical Appliances Plant, Guangdong, China) with a constant temperature of 25 °C and different daylengths approaching the critical daylengths of each population to produce non-diapausing and diapausing individuals (Table 1). Each experimental population was tested by rearing four to six replicates with at least 50 larvae per replicate. For all non-diapausing larvae, when larvae developed to the fifth instar, they were moved individually into cell culture plates with 12 holes (diameter: 2.4 cm; height: 2 cm for each hole) for checking the time of pupation and adult eclosion, and the developmental time from hatching to pupation and adult eclosion was recorded. For all diapausing larvae at 40 days of age (non-diapausing larvae generally pupated within 40 days after hatching at 25 °C in this experiment), they were individually transferred to plastic tubes with moist cotton balls and placed in a dark refrigerator of 5 °C for hibernation. After 92 days of dormancy, they were transferred to L:D 18:6, 25 °C to terminate diapause, and the time of pupation and adult emergence were recorded in detail.

The pupal weight on the 2nd day after pupation for both non-diapausing and diapausing individuals was calculated by using an electric balance (AUY120 produced by SHIMADZU Corporation, Kyoto, Japan). Growth rate was computed as ln pupal weight/larval developmental times [16]. Sexual size dimorphism (SSD), in which SSD = (size of the larger sex/size of the smaller sex) − 1 was estimated for both pupae and adults using the Lovich and Gibbons [46] index. For all populations, sexing was performed on the day of adult emergence. All newly emerged adults were moved individually to a plastic box (diameter: 2.5 cm; height: 3.5 cm) after the elimination of the meconium and weighed by using an electric balance. The proportionate weight loss from pupation to adult eclosion was figured using the formula: weight lost = l − (adult weight/pupal weight).

### 2.3. Statistical Analyses

The SPSS 19.0 (SPSS Inc., IBM, Armonk, NY, USA) was used for statistical analyses. The linear mixed model was used to analyze the life-history traits, in which population, sex and development pathway were regarded as fixed main factors and rearing box was regarded as a random effect. The full model containing all possible interaction terms was first executed, and the nonsignificant three-way term (population-by-sex-by-developmental-pathway) was removed from the final model in this analysis.

The differences in life-history traits among different geographic populations were analyzed using the one-way ANOVA test. The independent samples T-test (T test) was used to compare the differences in different developmental paths of each geographic population. One-way analysis of variance (ANOVA) and Duncan’s test were used to estimate sex differences in life history traits across developmental pathway and groups.

Linear regression analysis was used to calculate the correlations between developmental time and body weight. A binomial distribution test following a nonparametric test was used to calculate sex ratios.

## 3. Results

### 3.1. Developmental Pathway and Sex Ratio in Different Geographic Populations

When larvae from different geographic populations were reared at 25 °C under different daylengths, part of their populations entered diapause as fully grown larvae, while the rest developed to pupae without diapause (Table 1). A total of 43.9% to 59.4% of individuals from the SY, HB, JX and HF populations entered diapause, showing that the selected daylengths were close to their own critical daylengths. The percentage of diapause was 74.6% in the LF population and 28.9% in the TA population, suggesting that the selected daylength was slightly longer for the LF population and was slightly shorter for the TA population. The sex ratio was different for different populations. The proportion of females in the northern HB population was significantly higher (0.66 for non-diapausing individuals and 0.70 for diapausing individuals). No significant deviation from 0.50 was found in non-diapausing individuals of the SY population, but it showed a significantly lower female ratio of 0.40 for diapause-developing individuals. There was no significant deviation from 0.50 in the LF and TA populations. The HF population showed a significantly higher female ratio of 0.60 for both non-diapausing and diapausing individuals. The YX population showed a significantly lower female ratio of 0.37 for diapausing individuals.

### 3.2. Developmental Time

**Figure 2:**
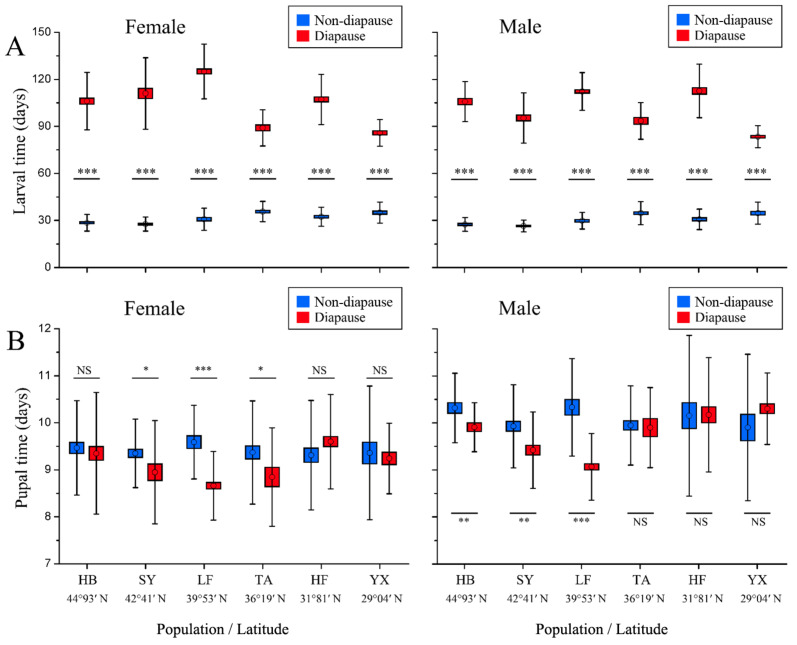
Larval (**A**) and pupal (**B**) developmental time for non-diapausing and diapausing individuals in different geographical populations of *Ostrinia furnacalis*. The asterisk indicates a significant difference between non-diapausing and diapausing individuals in each subgraph (* *p* ≤ 0.05, ** *p* ≤ 0.01, *** *p* ≤ 0.00). NS = Nonsignificant. Error bars indicate SE.

The larval developmental time was significantly affected by population, developmental pathway and their interaction (population × developmental pathway) (Table 2). The developmental time for non-diapausing individuals was significantly shorter than that for diapausing individuals in all populations for both females and males (Figure 2A). The larval developmental time for non-diapausing females and males tended to increase as latitude declined, with the middle-latitudinal TA population being longest. The larval developmental time in the low-latitudinal YX population was significantly longer than those in the high-latitudinal HB and SY populations (*p* < 0.05) (Figure 2A, Table S1) The larval developmental time for diapausing individuals showed clear peaks and troughs, with the lowest-latitudinal (YX) population being shortest, significantly shorter than those from the high-latitudinal HB and SY populations. For non-diapausing individuals, there was no significant difference in larval developmental time of females and males in all populations (*p* > 0.05). For diapausing individuals, female larval developmental time was significantly longer than male larval developmental time in the northern SY and LF populations. However, the developmental time of male larvae was significantly longer in males than that of female larvae in the southern TA and HF populations. There was no significant difference in larval developmental time between sexes in the HB and YX populations (Table S1).

The developmental time of pupae was significantly affected by sex (Table 2). Pupal developmental time in both non-diapausing and diapausing individuals did not show a constant latitudinal gradient (Figure 2B). Except for the diapausing individuals from the SY and LF populations, significant differences were found between the sexes for all other populations, with significantly longer developmental times in males than females (Table S1).

### 3.3. Pupal Weight and Growth Rate

**Figure 3:**
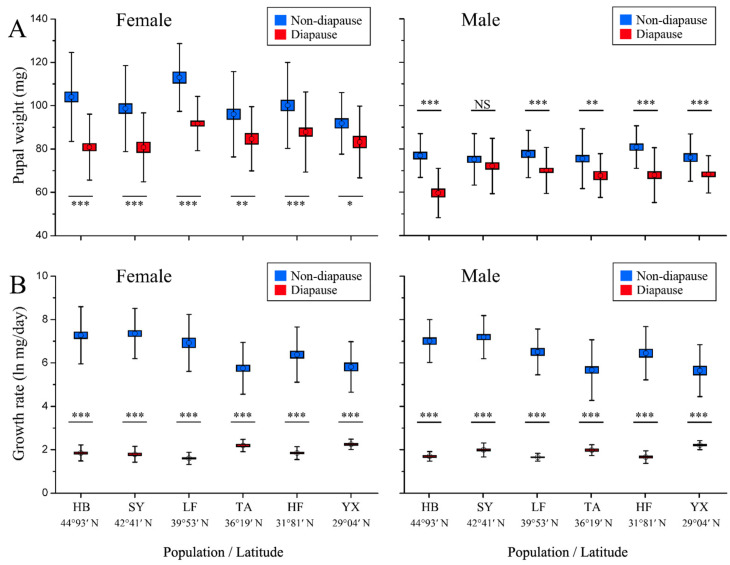
Pupal weight (**A**) and larval growth rate (**B**) for non-diapausing and diapausing individuals in different geographical populations of *Ostrinia furnacalis*. The asterisk indicates a significant difference between non-diapausing and diapausing individuals in each subgraph (* *p* ≤ 0.05, ** *p* ≤ 0.01, *** *p* ≤ 0.001). NS = Nonsignificant. Error bars indicate SE.

Pupal weight was significantly affected by population, developmental pathway, sex and their interactions (population × sex; population × developmental pathway; sex × developmental pathway; population × sex × developmental pathway) (Table 2). For both non-diapausing and diapausing individuals, pupal weight did not show a constant latitudinal gradient, with clear peaks and troughs, suggesting a sawtooth pattern (Figure 3A) The pupal weight of non-diapausing females from the middle-latitudinal LF population was largest, followed by the HB, HF, SY, and TA populations, with the low-latitudinal YX population being smallest. The pupal weight of diapausing females from the middle-latitudinal LF population was the largest, followed by the HF, TA and YX populations, with the higher-latitudinal SY and HB populations being smallest. Interestingly, the body weight of non-diapausing females was significantly higher in the northern HB population than in the southern YX populations; in contrast, diapausing females of the HB population had a smaller pupal weight than the YX population. Pupal weight was significantly larger in non-diapausing individuals than in diapausing individuals in all populations except for the males of the SY population (Figure 3A) Across all populations, pupae of females were significantly larger than that of males (Table S1).

Larval growth rate was significantly affected by population, developmental pathway, sex and their interactions (population × developmental pathway) (Table 2). In the non-diapausing individuals, the growth rate tended to slow down as latitude decreased, whereas the growth rate in diapausing individuals appeared to increase as latitude decreased (Figure 3B). In the non-diapausing individuals, the growth rate was significantly higher in the high-latitudinal HB and SY populations than in the low-latitudinal LF, TA, HF and YX populations for both females and males (F_23,1586_ = 559.325, *p* < 0.001). In the diapausing individuals, the growth rate of the low-latitudinal YX population was significantly higher than that of the high-latitudinal HB population for both sexes (*p* < 0.001). The growth rate was significantly lower in diapausing individuals than in non-diapausing individuals for all populations (*p* < 0.001, see Figure 3B). There were no significant differences in growth rate between females and males for either developmental pathway except for the LF populations for non-diapausing individuals (Table S1).

### 3.4. Adult Weight and Weight Loss

**Figure 4:**
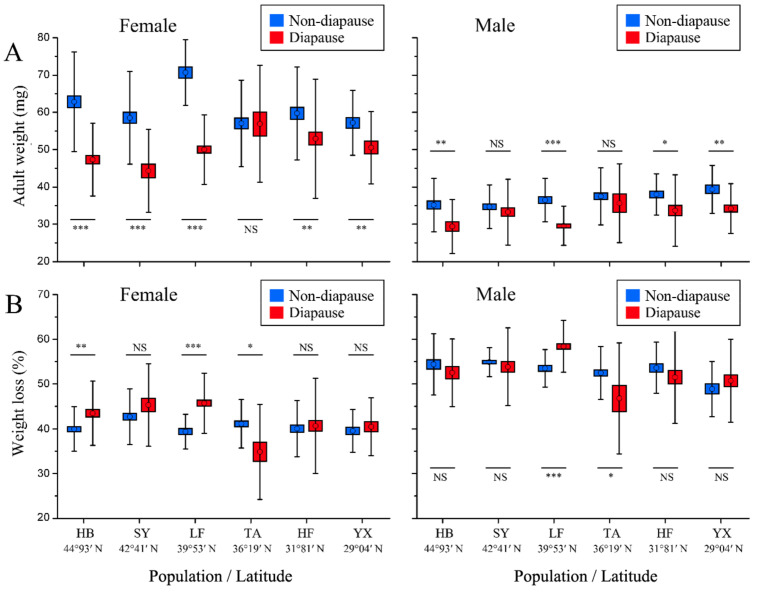
Adult weight (**A**) and weight loss (**B**) of non-diapausing and diapausing individuals in different geographical populations of *Ostrinia furnacalis*. The asterisk indicates a significant difference between non-diapausing and diapausing individuals in each subgraph (* *p* ≤ 0.05, ** *p* ≤ 0.01, *** *p* ≤ 0.001). NS = Nonsignificant. Error bars indicate SE.

Adult weight was significantly influenced by population, developmental pathway, sex and their interactions (population × sex; population × developmental pathway; sex × developmental pathway; population × sex × developmental pathway) (Table 2). Adult weight in both non-diapausing and diapausing females showed peaks and troughs without clear patterns, suggesting a sawtooth pattern (Figure 4A). The adult weight of non-diapausing females from the middle-latitudinal LF population was largest, followed by the HB, HF, SY, and TA populations, with the low-latitudinal YX population being smallest (Figure 4A, Table S1). Similar to the pupal weight, the adult weight of non-diapausing females was significantly higher in the northern HB population than in the southern YX population, whereas the adult weight of diapausing females was smaller in the northern HB population than in the southern YX population. It is interesting to note that the adult weight of non-diapausing males tended to increase as latitude decreased, following the converse Bergmann rule. However, the adult weight of diapausing males varied greatly among populations, with the TA population being largest. In all populations, the body weight of female adult of non-diapausing individuals was significantly greater than that of diapausing individuals (*p* < 0.01, see Figure 4A) except for the TA population. Male adult weight of non-diapausing individuals was larger than that of diapausing individuals, with significant differences in the HB, LF, HF and YX populations. Between the sexes, female adults were significantly larger than males for all populations (Table S1).

Weight loss was significantly affected by population, developmental pathway, sex and their interactions (population × developmental pathway; sex × developmental pathway) (Table 2). Weight loss was significantly different among populations (F_23, 1278_ = 46.617, *p* < 0.001). In both developmental pathways in all populations, male pupae lost significantly more body weight at metamorphosis than females (Table S1). Except for females of the HB, LF and TA populations and the LF and TA males (with more weight loss in diapausing individuals than non-diapausing individuals for the HB and LF populations and less for the TA population for both sexes), there were no significant differences in weight loss between the two developmental pathways (Figure 4B).

### 3.5. Sexual Size Dimorphism

**Figure 5:**
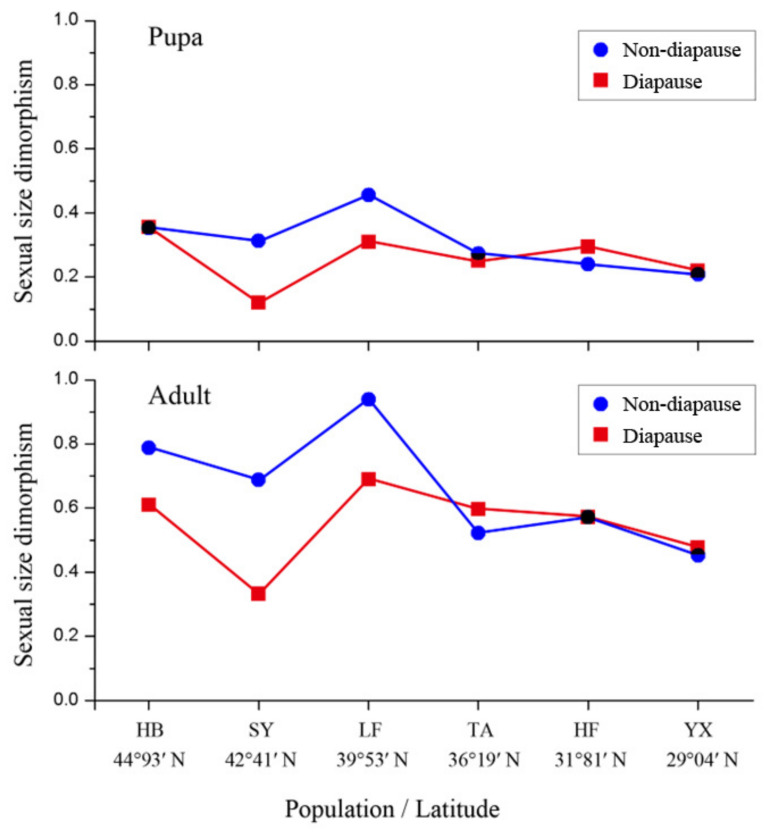
Sexual size dimorphism for non-diapausing and diapausing individuals in different geographical populations of *Ostrinia furnacalis*.

The sizes of SSD for pupae and adults are revealed in Figure 5. The SSD of pupae and adults from the LF population was largest, while the SY population was the smallest. The SSD in the adult stage was greater than that of the pupal stage, regardless of the developmental pathway. In the SY and LF populations, the SSD was larger in non-diapausing individuals than in diapausing individuals.

### 3.6. Relationship between Larval Developmental Time and Pupal Weight

**Figure 6:**
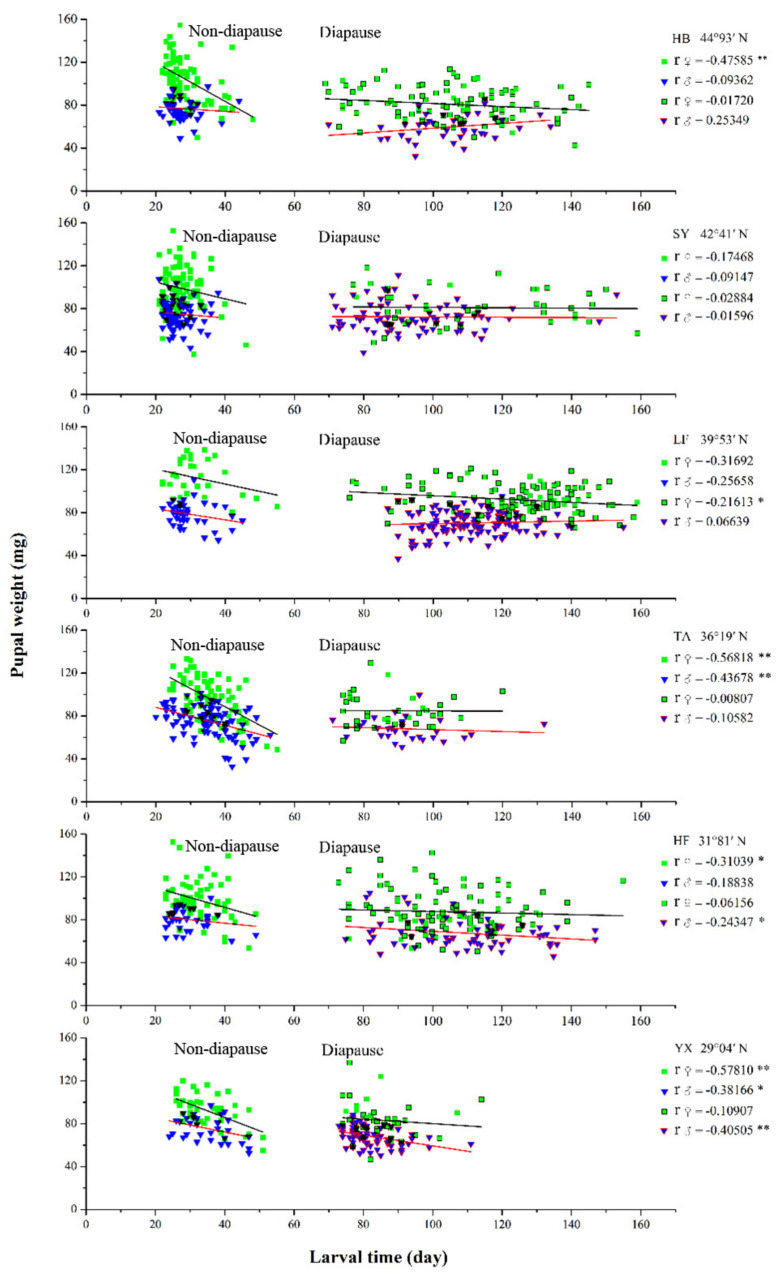
Relationships between larval developmental time and pupal weight for non-diapausing and diapausing individuals in different geographical populations of *Ostrinia furnacalis*. The asterisk indicates a significant correlation existed between larval developmental time and pupal weight (* *p* ≤ 0.05, ** *p* ≤ 0.01).

In the non-diapausing individuals, pupal weight tended to decrease as larval developmental time increased (Figure 6). A significant negative relationship was found between pupal weight and larval developmental time in the HB female population (*p* < 0.01), the TA population (*p* < 0.01 for both female and male), the HF female population (*p* < 0.05) and the YX population (*p* < 0.01 for female, *p* < 0.05 for male). In diapausing individuals, a weak negative relationship between pupal weight and larval developmental time was found in all populations (*p* > 0.05), except for the HB male population (a weak positive relationship) and the HF and YX male populations (a significant negative relationship, *p* < 0.05 for the HF population, *p* < 0.01 for the YX population).

## 4. Discussions

To the best of our knowledge, the current study represents the first attempt to examine the differences in life-history traits between non-diapausing and diapausing individuals in an insect species along a latitudinal gradient. The presentation of the two developmental pathways in Table 1 reveals the latitudinal-gradient variation in the critical daylength of diapause induction in *O. furnacalis*. Consistent with our previous observations [42], the critical daylength increased with increasing latitude, showing geographically based differences in the propensity to continue development or enter diapause. The positive correlation between the critical daylength and latitude indicates that there is local adaptation in the development pattern, similar to what has been found in some insect species, such as the water strider, *Aquarius remiges* [47,48], the butterfly, *Arcia agestis* [49], the parasitoid, *Nasonia vitripennis* [50], and the fall webworm, *Hyphantria cunea* [51]. The expression of different developmental pathways in each population also suggests that each individual has its own photoperiodic response threshold. Those individuals, above a certain threshold, can enter diapause; those below it can avoid diapause. In other words, different developmental pathways may result in conditionally expressed genes. Although they are carried by all individuals in a population, only a fraction of these individuals are expressed and exposed to selection at any given time [52]. Understanding the expression of different developmental pathways may be vital for understanding life-history evolution [53].

The most interesting result of our study is that the latitudinal variations significantly affect different life-history traits of *O. furnacalis*, including non-diapausing or diapausing, developmental time, body weight and growth rate. In the non-diapausing pathway, the larval developmental time was shorter and the growth rate was higher in the high-latitudinal populations than that in the low-latitudinal populations, following countergradient variation (termed “antagonistic selection” [54]), i.e., the genetic and environmental influences on phenotypes are opposed to each other, thereby reducing the phenotypic differentiation between populations [31]. In the diapausing pathway, the high-latitudinal populations had longer larval developmental times and lower growth rates than the low-latitudinal populations (Figure 2A and Figure 3B), suggesting latitudinal cogradient variation, i.e., the effects of genetics and environment on phenotypic expression are consistent across traits (called “synergistic selection,” [54]); thus, phenotypic variation is emphasized in populations across the gradients [31]). Those non-diapausing individuals in high-latitudinal populations have shorter developmental times and higher growth rates, which should be related to the length of the growing season. Because high latitudes are usually associated with colder winters and shorter growing seasons, such conditions require insects to grow rapidly, completing an entire generation in a shorter growing season or resulting in some individuals developing directly to the adult stage, producing a second generation in summer before the diapause-inducing photoperiod approaches. For example, the high-altitudinal HB population is dominated by univoltine. Larvae developed in summer experienced a gradually shortened photoperiod and almost all larvae entered winter diapause. The few larvae that hatch at the end of June can develop without diapause and result in a second generation in mid-August [41]. The diapausing populations showed latitudinal cogradient variation, which should be linked to winter climatic conditions because high- and low-latitudinal populations experience completely different climatic conditions. Winters where high latitudinal populations occur are extremely cold; for example, the average daily minimum temperature in Harbin city (44°93′ N, 127°17′ E) is below minus 20 °C. Conversely, winters are mild at low latitudes; for example, the average daily minimum temperature in Yongxiu County (29°04′ N, 115°82′ E) is about 5 °C in January, the coldest month. Therefore, the hibernating populations in high latitudes experience strong selection under very low winter temperatures and long winter conditions, thus resulting in longer diapause duration; the low-latitudinal populations experience mild winter temperature and short winter conditions, hence exhibiting shorter diapause duration. We suspect that the difference in the duration of diapause of *O. furnacalis* between high- and low-latitudinal populations may be genetically based, which needs to be confirmed in future research.

Additionally, different models of body weight were also found between the two alternative developmental pathways. The northern HB population displayed significantly higher body weight than the southern YX population in the non-diapausing pathway, suggesting a countergradient variation, whereas the body weight in the diapause pathway was slightly smaller in the northern HB population than in the southern YX population, without showing gradient variation. However, the reason for the difference in body weight between non-diapausing and diapausing populations remains unclear. It is worth mentioning that the high-latitudinal HB and SY females achieved significantly higher body weight than did the low-latitudinal YX females in the non-diapausing pathway, even though the developmental time of the high-latitudinal HB and SY females was significantly shorter than that of the low-latitudinal YX females, indicating that there is genetic variation in developmental time and body weight among populations. Therefore, larval developmental time is not necessarily correlated with body weight. A similar situation also occurred in the cabbage beetle *C. bowringi*, where a longer larval developmental time resulted in a small body weight in high-latitudinal populations, whereas a shorter larval developmental time resulted in a large body weight in low latitudinal populations [33]. This study also showed that body weight of both pupae and adults from non-diapausing individuals was significantly larger than those from diapausing individuals in almost all populations, suggesting that diapause has a cost. This trade-off is likely caused by the loss of metabolic reserves during diapause maintenance [47,55].

Our study indicates that weight loss between non-diapausing and diapausing individuals varied among populations, but it did not show a regular change. However, in all populations, male pupae lost significantly more weight during metamorphosis than females, suggesting that males either hold a higher metabolic rate than females or are less effective at preventing water loss [56]. Consistent with a previous study [34], the body weight of *O. furnacalis* was significantly larger in females than males, and the SSD values were lower in the pupal stage than in the adult stage in all populations, which suggested that weight loss played an important role in the regulation of SSD [57].

The relationship between larval developmental time and pupal weight was also different between the two different developmental pathways. A negative relationship was found in the non-diapausing individuals between the two traits, with significant differences in most populations, whereas the diapausing individuals showed no relationship or a significantly negative relationship in a few populations. This study first reveals that the relationship between larval developmental time and pupal weight varies depending on the developmental pathways.

## 5. Conclusions

Although latitudinal variation in life-history traits for both non-diapausing and diapausing individuals of *O. furnacalis* showed a saw-tooth pattern, we found that life-history patterns of the two alternative developmental pathways were significantly different between the lower and higher latitudes. In the non-diapausing pathway, the larval development time and growth rate showed countergradient variation, whereas the larval development time and growth rate showed cogradient variation in the diapausing pathway. In the non-diapausing pathway, body weight showed a countergradient variation, whereas the body weight in the diapausing pathway did not show a gradient variation. Furthermore, shorter larval development in the non-diapausing pathway was associated with higher body weight in the high latitudinal populations, whereas longer larval development time was associated with smaller body weight in the low latitudinal populations. The non-diapausing individuals were significantly larger than the diapausing individuals in almost all populations, suggesting that extending diapause is more costly. The relationship between larval development time and pupal weight was also different between the two alternative developmental pathways. To our knowledge, this is the first study revealing latitudinal variation in the life-history traits of *O. furnacalis* for both non-diapausing and diapausing individuals. These results provide new insights into the evolution of life-history traits in this moth.

## Figures and Tables

**Table 1:** Incidence of development pathways and sex ratio in six different populations of *Ostrinia furnacalis* when larvae were reared at 25 °C under different daylengths that were close to their critical daylengths.

Population	Geographic Position	Rearing Daylength (h)	Developmental Pathway	Sex	N	Female %	Diapause %
HB	44°93′ N 127°17′ E	15.5	Non-diapause	♀	87	65.9 *	50.6
				♂	45		
			Diapause	♀	94	69.6 *	
				♂	41		
SY	42°41′ N 123°29′ E	15.0	Non-diapause	♀	85	52.5	43.9
				♂	77		
			Diapause	♀	50	39.4 *	
				♂	77		
LF	39°53′ N 116°68′ E	14.5	Non-diapause	♀	40	46.0	74.6
				♂	47		
			Diapause	♀	127	49.6	
				♂	129		
TA	36°19′ N 117°09′ E	14.5	Non-diapause	♀	74	44.3	28.9
				♂	93		
			Diapause	♀	38	55.9	
				♂	30		
HF	31°81′ N 117°22′ E	14.0	Non-diapause	♀	70	59.8 *	59.4
				♂	47		
			Diapause	♀	103	60.2 *	
				♂	68		
YX	29°04′ N 115°82′ E	13.5	Non-diapause	♀	46	54.8	57.6.
				♂	38		
			Diapause	♀	42	36.8 *	
				♂	72		

By a binomial test, the asterisk indicates a significant difference from 0.5 (*p* < 0.05) between sexes. HB: Harbin city, Heilongjiang Province; SY: Shenyang city, Liaoning Province; LF: Langfang city, Hebei Province; TA: Taian city, Shandong Province; HF: Hefei city, Anhui Province; YX: Yongxiu County, Jiangxi Province.

**Table 2:** The results of the linear mixed model analyses of larval time, pupal time, pupal weight, growth rate, adult weight and weight loss of *Ostrinia furnacalis* depended on population, sex, and development pathway (dvpt. path.).

Traits	Fixed Effects	*df*	*F*	*p*
Larval time	Population	5	11.051	**<0.001**
	Sex	1	0.517	0.472
	Dvpt. path.	1	1411.443	**<0.001**
	Population × Sex	5	3.633	**0.003**
	Population × Dvpt. path.	5	21.820	**<0.001**
	Sex × Dvpt. path.	1	3.777	0.052
	Population × Sex × Dvpt. path.	5	8.850	**<0.001**
Pupal time	Population	5	1.092	0.363
	Sex	1	13.882	**<0.001**
	Dvpt. path.	1	2.617	0.106
	Population × Sex	5	1.197	0.308
	Population × Dvpt. path.	5	1.715	0.128
	Sex × Dvpt. path.	1	0.000	0.987
Pupal weight	Population	5	5.508	**<0.001**
	Sex	1	138.757	**<0.001**
	Dvpt. path.	1	79.143	**<0.001**
	Population × Sex	5	3.761	**0.002**
	Population × Dvpt. path.	5	3.550	**0.003**
	Sex × Dvpt. path.	1	15.726	**<0.001**
	Population × Sex × Dvpt. path.	5	3.053	**0.010**
Growth rate	Population	5	12.202	**<0.001**
	Sex	1	3.929	**0.048**
	Dvpt. path.	1	1134.056	**<0.001**
	Population × Sex	5	1.425	0.212
	Population × Dvpt. path.	5	9.663	**<0.001**
	Sex × Dvpt. path.	1	1.617	0.204
Adult weight	Population	5	10.024	**<0.001**
	Sex	1	261.309	**<0.001**
	Dvpt. path.	1	82.125	**<0.001**
	Population × Sex	5	6.272	**<0.001**
	Population × Dvpt. path.	5	8.500	**<0.001**
	Sex × Dvpt. path.	1	27.797	**<0.001**
	Population × Sex × Dvpt. path.	5	4.650	**<0.001**
Weight loss	Population	5	2.227	**0.049**
	Sex	1	114.932	**<0.001**
	Dvpt. path.	1	5.892	**0.015**
	Population × Sex	5	1.467	0.198
	Population × Dvpt. path.	5	2.771	**0.017**
	Sex × Dvpt. path.	1	5.101	**0.024**

Note: The significant effects are shown in bold.

## Data Availability

Data are contained within the article.

## Supplementary Materials

The following supporting information can be downloaded at: https://www.mdpi.com/article/10.3390/insects13111026/s1, Table S1: Data of life history traits between non-diapause and diapause development in different geographical population of *Ostrinia furnacalis* at 25 °C.
