# Supplementary material for: Differentiation of Developmental Pathways Results in Different Life-History Patterns between the High and Low Latitudinal Populations in the Asian Corn Borer
Source: Insects. 2022 Nov 6;13(11):1026. doi: 10.3390/insects13111026 (PMC9696888; doi:10.3390/insects13111026)
Supplement: Supplementary file 1 [file insects-13-01026-s001.zip › insects-1948223-supplementary.pdf]

Table S1 Data of life history traits between non-diapause and diapause development in different geographical population of *Ostrinia furnacalis* at 25°C.

Population	Development pathway	Number	Larval time (day)	Pupal time (day)	Pupal weight (mg)	Growth rate ((LNmg)/day)	Adult weight (mg)	Weight loss (%)
HB	Non-diapause (females)	87	28.52 ± 0.57 jk	9.47 ± 0.12 cde	104.05 ± 2.23 b	7.28 ± 0.14 ab	62.83 ± 1.56 b	39.94 ± 0.59 i
	Non-diapause (males)	45	27.42 ± 0.65 jk	10.32 ± 0.11 a	76.94 ± 1.51 ij	7.01 ± 0.15 bc	35.15 ± 1.11 hi	54.41 ± 1.06 b
	Diapause (females)	94	106.12 ± 1.89 d	9.35 ± 0.15 def	80.86 ± 1.57 ghi	1.85 ± 0.04 gh	47.34 ± 1.13 fg	43.47 ± 0.83 gh
	Diapause (males)	41	105.83 ± 2.00 d	9.91 ± 0.09 abc	59.64 ± 1.80 m	1.70 ± 0.03 gh	29.41 ± 1.28 j	52.53 ± 1.34 bc
SY	Non-diapause (females)	85	27.64 ± 0.49 jk	9.35 ± 0.09 def	98.69 ± 2.15 bc	7.36 ± 0.13 a	58.54 ± 1.47 bc	42.70 ± 0.74 ghi
	Non-diapause (males)	77	26.49 ± 0.43 k	9.93 ± 0.11 abc	75.18 ± 1.35 ijk	7.19 ± 0.11 abc	34.69 ± 0.71 hi	54.91 ± 0.40 b
	Diapause (females)	50	111.02 ± 3.23 bc	8.95 ± 0.18 fgh	80.79 ± 2.27 ghi	1.79 ± 0.05 gh	44.32 ± 1.81 g	45.32 ± 1.49 fg
	Diapause (males)	77	95.38 ± 1.83 e	9.42 ± 0.10 def	72.12 ± 1.45 jkl	1.99 ± 0.04 fg	33.27 ± 1.16 ij	53.84 ± 1.14 bc
LF	Non-diapause (females)	40	30.80 ± 1.12 hijk	9.59 ± 0.13 bcd	113.02 ± 2.50 a	6.92 ± 0.21 c	70.69 ± 1.54 a	39.38 ± 0.68 i
	Non-diapause (males)	47	29.79 ± 0.77 ijk	10.33 ± 0.17 a	77.69 ± 1.59 hij	6.51 ± 0.15 d	36.48 ± 0.93 hi	53.52 ± 0.67 bc
	Diapause (females)	127	125.05 ± 1.55 a	8.66 ± 0.07 h	91.75 ± 1.11 de	1.60 ± 0.02 h	50.02 ± 0.91 ef	45.72 ± 0.66 fg
	Diapause (males)	129	112.29 ± 1.06 b	9.07 ± 0.07 efgh	70.07 ± 0.94 kl	1.66 ± 0.02 gh	29.59 ± 0.52 j	58.40 ± 0.58 a
TA	Non-diapause (females)	74	35.64 ± 0.76 h	9.37 ± 0.14 def	96.05 ± 2.31 cd	5.75 ± 0.14 e	57.05 ± 1.45 cd	41.11 ± 0.68 hi
	Non-diapause (males)	93	34.67 ± 0.77 hi	9.95 ± 0.10 ab	75.50 ± 1.43 ijk	5.67 ± 0.15 e	37.49 ± 0.88 hi	52.47 ± 0.69 bc
	Diapause (females)	38	89.00 ± 1.87 f	8.85 ± 0.21 gh	84.71 ± 2.40 fg	2.19 ± 0.05 f	56.91 ± 3.20 cd	34.86 ± 2.17 j
	Diapause (males)	30	93.50 ± 2.15 e	9.90 ± 0.19 abc	67.67 ± 1.85 l	1.98 ± 0.05 fg	35.66 ± 2.49 hi	46.81 ± 2.92 ef
HF	Non-diapause (females)	70	32.36 ± 0.73 hij	9.31 ± 0.15 def	100.12 ± 2.38 bc	6.39 ± 0.15 d	59.74 ± 1.61 bc	40.04 ± 0.81 i
	Non-diapause (males)	47	30.72 ± 0.95 hijk	10.15 ± 0.27 a	80.83 ± 1.43 ghi	6.45 ± 0.18 d	38.02 ± 0.89 hi	53.63 ± 0.92 bc
	Diapause (females)	103	107.20 ± 1.58 cd	9.60 ± 0.11 bcd	87.86 ± 1.82 ef	1.85 ± 0.03 gh	52.95 ± 1.72 de	40.66 ± 1.15 hi
	Diapause (males)	68	112.63 ± 2.07 b	10.17 ± 0.17 a	67.92 ± 1.54 l	1.66 ± 0.03 gh	33.70 ± 1.38 ij	51.50 ± 1.49 bcd
YX	Non-diapause (females)	46	34.98 ± 0.99 h	9.36 ± 0.23 def	91.84 ± 2.12 de	5.82 ± 0.17 e	57.21 ± 1.41 cd	39.53 ± 0.78 i
	Non-diapause (males)	38	34.66 ± 1.14 hi	9.90 ± 0.28 abc	76.01 ± 1.77 ijk	5.65 ± 0.19 e	39.37 ± 1.16 h	48.89 ± 1.11 de
	Diapause (females)	42	85.81 ± 1.31 fg	9.24 ± 0.13 defg	83.25 ± 2.55 fgh	2.25 ± 0.04 f	50.57 ± 1.69 ef	40.47 ± 1.12 hi
	Diapause (males)	72	83.42 ± 0.83 g	10.30 ± 0.10 a	68.24 ± 1.02 l	2.21 ± 0.02 f	34.22 ± 0.93 i	50.74 ± 1.29 cd

Larval time: df =23,1594, F =730.230, $P < 0.001$; **Pupal time:** df =23,1366, F =13.636, $P < 0.001$; **Pupal weight**: df =23,1588, F =49.444, $P < 0.001$; **Growth rate**: df =23,1586, F =559.325, $P < 0.001$; **Adult weight**: df =23,1279, F =75.800, $P < 0.001$; **Weight loss**: df =23,1278, F =46.617, $P < 0.001$.

Note: all means are given with Mean ± Standard error (SE).
